# The effectiveness of mindfulness yoga on patients with major depressive disorder: a systematic review and meta-analysis of randomized controlled trials

**DOI:** 10.1186/s12906-023-04141-2

**Published:** 2023-09-08

**Authors:** Chuyuan Miao, Yun Gao, Xiaohua Li, Ying Zhou, Joanne Wai-yee Chung, Graeme D. Smith

**Affiliations:** 1 School of Nursing, Guangzhou Medical University, 195, Dongfengxi Road, Yuexiu District, Guangzhou, Guangdong Province 510182 China; 2 Hong Kong Nang Yan College of Higher Education, 325-329 Lai Chi Kok Road, Sham Shui Po, Kowloon, Hong Kong SAR 999077 China; 3 School of Health Sciences, Caritas Institute of Higher Education, Tseung Kwan O, Hong Kong SAR 999077 China

**Keywords:** Yoga, Mindfulness, Depression, Major depressive disorder, Meta-analysis, Systematic review

## Abstract

**Background:**

Mindfulness yoga is a type of exercise that emphasizes the integration of mindfulness or meditation into yoga. The aim of this study was to determine the effectiveness of mindfulness yoga intervention on major depressive disorder (MDD) patients.

**Methods:**

A systematic review and meta-analysis of randomized controlled trials (RCTs) was conducted by searching nine databases, including PubMed, EMBASE, Web of Science, The Cochrane Library, MEDLINE, China National Knowledge Infrastructure (CNKI), Wanfang Data knowledge service platform, Chinese Biomedical Literature Database (CBM), and China Science and Technology Journal Database (VIP) from inception to April 2023. Primary outcomes included the severity of depression. Secondary outcomes included anxiety and rumination.

**Results:**

Nine RCTs met our inclusion criteria (n = 581). The meta-analysis showed that mindfulness yoga significantly has a significant effect on depression (SMD = -0.53; 95%CI = -0.96 to -0.11; *P* < 0.05) among MDD patients. The only two RCTs involved also showed that mindfulness yoga could alleviate the anxiety level of MDD patients after intervention (SMD = -1.08; 95%CI = -1.64 to -0.52; *P* < 0.05). Meta-analysis did not reveal positive effects of the mindfulness yoga groups on rumination after intervention based on three RCTs (SMD = -0.33; 95%CI = -0.89 to 0.23; *P* > 0.05), but found a significant difference in the follow-up period based on two RCTs (MD = -7.42; 95%CI = -11.27 to -3.56; *P* < 0.05), compared with the control groups.

**Conclusion:**

Although we were unable to provide conclusive evidence to support the effectiveness of mindfulness yoga in improving symptoms in MDD patients, we found the literature included in this study indicated that mindfulness yoga might have a potential benefit for MDD patients and should be a feasible, acceptable, and promising intervention.

**Supplementary Information:**

The online version contains supplementary material available at 10.1186/s12906-023-04141-2.

## Highlights

Mindfulness yoga is a promising intervention for MDD patients.

Larger sample size and well-designed RCTs are needed for future studies.

Mindfulness yoga intervention, such as the form, intensity and period, require further clarification in the future studies.

**Supplementary Information:**

The online version contains supplementary material available at 10.1186/s12906-023-04141-2.

## Background

Major depressive disorder (MDD), also called depression, is the most common type of depressive disorder and a clinical syndrome mainly manifested by persistent depression with physical and mental symptoms [1]. According to 2015 statistics from the World Health Organization, about 322 million people worldwide suffer from depression, equivalent to 4.4% of the world’s population [2].

From 2010 to 2018, the number of adults with MDD in the United States increased from 15.5 to 17.5 million, while the proportion of adults aged 18 to 34 with MDD rose from 34.6–47.5% [3]. WHO has projected that this disease will rank first by 2030 [4]. In addition, as depression is the leading cause of suicide, the number of deaths due to depression is as high as 788,000 per year [5].

Depression affects patients’ quality of life and causes an enormous burden for society and families regarding health care costs and long-term treatment [6]. The financial burden of adults with MDD in the United States reportedly increased by 37.9% from 2010 to 2018, from $236.6 billion to $326.2 billion [3]. Thus, improving these patients’ physical and mental health can significantly impact their quality of life. In addition to pharmacotherapy, psychological therapy, and exercise have become increasingly more widely accepted because they can possibly produce the same effect for alleviating depressive symptoms without the risk of unpleasant side effects [7, 8].

Cognitive Behavioral Therapy (CBT) is recommended as the first-line psychological treatment for mild to severe MDD by the Agency for Healthcare Research and Quality (AHRQ) [9]. And as the third wave of CBT, mindfulness-based interventions (MBIs) provide a promising psychotherapeutic strategy for MDD [10]. Mindfulness is a technique that requires individuals to accept the present moment with non-judgment, tune out distractions, and focus exclusively on their breathing [11, 12]. Additionally, research has shown that mindfulness meditation may improve aerobic physical activity because of its self-regulatory capacities [13]. A previous research found that mindfulness-based therapy can prevent the recurrence of major depression [14]. Evidence also suggests that mindfulness-based therapy can help MDD patients’ self-awareness, control attention and regulate emotion by regulating the prefrontal cortex, cingulate cortex, and basal ganglia [15]. A meta-analysis based on 39 studies totaling 1140 participants suggests that mindfulness-based therapy is a promising intervention for treating anxiety and mood problems in the clinical population [16]. Another meta-analysis suggests that mindfulness-based interventions were superior to the comparison group and equivalent to evidence-based treatments for depression [17]. Therefore, MBIs are a promising psychotherapeutic strategy for MDD.

Besides, it is known that individuals with depressive symptoms frequently have more sedentary lifestyles. Yoga, an exercise with cost-effective and manageable characteristics, has also been suggested as a positive way to improve the overall well-being of MDD patients [18, 19]. Yoga meditation may modulate the sympathetic and limbic system activity [20]. It is believed that yoga asanas are related to the neurohumoral regulation system and impact neurotransmitter systems and the immune system, which may help with physical or cognitive symptoms of depression, stress, and anxiety [21]. Although there is limited evidence to support the use of yoga as an adjunctive treatment for MDD, it has been proposed as an alternative approach to reduce depressive symptoms.

Furthermore, there is evidence to support the use of a combination of mindfulness and yoga in those with depression [22–27]. Mindfulness yoga, a form of physical and mental activity used to manage emotions, is regarded to potentially impact the treatment of depressive and anxious symptoms [28]. Some researchers found that combining yoga with elements of mindfulness is more likely to draw positive outcomes than conventional treatment approaches [29, 30]. The investigators also revealed that the neuroplasticity of biomarkers was associated with the degree of depression, and there was a significant increase in brain-derived neurotrophic factor (BDNF) after mindfulness yoga intervention [31]. Although neurobiological effects remain unclear, yoga and meditation have been demonstrated to improve neuroplasticity in the brain of yoga practitioners with neuropsychiatric conditions [21, 32]. Therefore, the combination of mindfulness and yoga may provide an effective method for MDD sufferers to improve their health status.

Mindfulness Yoga is a synthesis of mind-body and spiritual exercise, first introduced in 1979 by Professor Kabat-Zinn and used in mindfulness-based stress intervention [33]. Mindfulness yoga focuses on the connection between consciousness and body, as well as noticing and accepting the present moment in the physical and mental experience [34]. It places a focus on posture adjustment and mind-body awareness, involving basic yoga techniques like controlled breathing, meditative or mindfulness techniques, and simple physical postures [35–37]. Current research on mindfulness yoga often adds elements of mindfulness-based therapy that have included body scans, standing or sitting posture training, and mindful walking [36–38]. Additionally, mindfulness yoga has experience-sharing for each participant. The whole process is usually under the guidance of one or several professional teachers [38, 39]. Moreover, mindfulness yoga differs from other forms of yoga styles because of its strong emphasis on extensive meditation practices, breathing exercises, yoga philosophy, and the informal application of a mindful awareness in daily life [39]. For now, there is no internationally accepted definition of mindfulness yoga, also regarded by scholars as mindfulness-based yoga or mindful yoga [29, 40]. For the purpose of this review, mindfulness yoga is defined as the combination of mindfulness or meditation with yoga, with no restrictions placed on the type of yoga.

Several systematic reviews have evaluated the effect of yoga, highlighting its significant impact on reducing depression [26, 41, 42]. Systematic reviews have shown that mindfulness yoga may be a determinant of improving the physical health of women [43, 44]. Cramer et al. (2017) found that yoga was more effective than exercise and medication in treating depression. The study integrated seven randomized controlled trials, which included a total of 240 MDD participants [23]. Additionally, Wu et al. (2023) also paid attention to studies on the effect of yoga on MDD patients. They included 34 literatures, focusing on indicators of depression and anxiety, and found that yoga improved depression symptoms and anxiety levels in MDD patients in the short term [45]. But to our knowledge, the impact of mindfulness yoga on the mental health of patients with major depressive disorder has yet to be systematically assessed. Hence, in our study, we attempted to explore the effectiveness of mindfulness yoga on MDD patients on rumination, depression and anxiety. We also tried to explore the stress and quality of life indicators based on the included RCTs. In addition, this study hopes to provide an evidence-based basis for further promotion and application of the treatment modality, for example by providing some indications for the implementation of mindfulness yoga for patients with major depression, and by providing comprehensive and integrated support for promoting collaboration between the multidisciplinary fields of medicine, kinesiology and education. Therefore, our study aims to systematically review and summarize data to evaluate the effects of mindfulness yoga on major depression disorder patients’ psychological health when compared to a non-limitation comparator arm.

## Methods

The methods we used for reviewing the literature and reporting the results of this study complied with the Preferred Reporting Items for Systematic Reviews and Meta-analysis (PRISMA) [46]. Our protocol was registered in PROSPERO (CRD42022315329).

### Inclusion and exclusion criteria

Full details of inclusion and exclusion criteria in this review is provided in Table 1.

**Table 1:** Inclusion and Exclusion Criteria

Criteria (PICOS)	Inclusion criteria	Exclusion criteria
Participants	(1) Study participants are at least 18 years old; (2) Major depressive disorder patients are included at least one of the following requirements: ①Diagnosed by the clinical doctor; ②Study participants must meet the inclusion criteria for major depressive disorder patients based on the Diagnostic and Statistical Manual of Mental Disorders, 4th/5th Edition (DSM-4/DSM-5) or the International Statistical Classification of Diseases 10th/11th Revision (ICD-10/ICD-11).	Participants who have psychiatric or physical diseases except for major depressive disorder.
Interventions	Interventions must be presented in detail and involve both meditation/mindfulness and yoga components: (1) Each Intervention must have the same parts and encompass; meditation/mindfulness, asanas (yoga posture), and pranayama (yoga breathing techniques); (2) Yoga is the main intervention that its movement component (physical activity); (3) Duration and frequency of the practice do not affect the Intervention’s eligibility; (4) All forms of intervention are included, like online/DVD/offline, etc.	Interventions may mention mindfulness or meditation practices, but the primary components, such as laughter yoga, are not mindfulness or meditation elements.
Comparison	There is no limitation on the comparator arm. It can be any other intervention type, like other psychological and physical interventions/waitlist controls/usual treatment, etc.	N/A
Outcomes	(1) Studies include at least one measurement of depression, anxiety, rumination; (2) All of them were measured by validated self-reported or clinician-administered scales (For example, Depression can be measured by the Beck Depression Inventory or the Hamilton Rating Scale; Anxiety can be measured by the Hamilton Anxiety Scale; Rumination can be measured by the Ruminative Responses Scale).	N/A
Study design	Only randomized controlled trials (RCTs) in English or Chinese were eligible.	(1) Duplicated studies; (2) Full text cannot be reached; (3) No data or not clear reported for analysis; (4) Unpublished literature; (5) Studies lacking comparison group; (6) RCTs registries or ongoing studies.
Setting	There were no restrictions on places. It could take place in any other places like hospitals, centers, homes, etc.	N/A

**Abbreviation: N/A, Not applicable**

### Search strategy

We searched nine databases, including PubMed, EMBASE, Web of Science, The Cochrane Library, Medline, China National Knowledge Infrastructure (CNKI), Wanfang Data knowledge service platform, Chinese Biomedical Literature Database (CBM), and China Science and Technology Journal Database (VIP) with no restrictions or limits applied. Search strategies for each database were customized and combined Medical Subject Headings term (MeSH) and Entry Terms to represent the concepts of major depressive disorder and mindfulness yoga. We developed an electronic search strategy using terms and free words. Our databases were searched using the following terms: Yoga, Mindful*, Meditation, Depression, Depressive disorder, Depressi*, randomized controlled trial, randomized clinical trial. Only RCTs were included within the review. Search terms were adapted, where necessary, for specific databases. We searched the following electronic databases from their start date until April 12th, 2023. In addition, we also maintain track of our searches for additional relevant references within the listed RCTs. After removing duplicate manuscripts, CYM and XHL screened paper titles and abstracts firstly to identify potentially eligible articles. Then two independent reviewers (CYM and XHL) assessed the eligibility of the full-text articles.

### Study selection

Two different researchers (CYM and XHL) independently screened all the titles and abstracts of all identified studies, studies meeting the inclusion criteria were then selected for the next stage. Researchers then independently selected full-text articles according to our inclusion and exclusion criteria. Following discussion between two researchers, articles that did not meet all the criteria and duplicates were excluded and removed. All extracted data was checked for consistency, and a third researcher (YG) to resolve any differences. The researchers’ inter-rater reliability were using Cohen’s Kappa with the SPSS version 25.0 tool. Full-text screening inter-rater reliability was moderate (Cohen’s Kappa = 0.417), *P* = 0.001 < 0.05, indicating that the two reviewers judged the results to be consistent.

### Data extraction

We (CYM and XHL) used an Excel spreadsheet to record pertinent extraction information. The researchers extracted the characteristics of included studies, including the basic features such as the first authors’ name/year, country, primary outcome measures, secondary outcomes measures, data collection instrument, intervention/duration/frequency, population/sample size [see Supplementary Table 2].

### Methodological quality assessment of each eligible study

To determine the risk of bias, the methodological quality of studies were assessed RCTs using version 2 of the Cochrane risk-of-bias tool for randomized trials (RoB 2) from The Cochrane Handbook for Systematic Reviews of Interventions [47]. The Excel tool we used to complete the ROB 2 assessments is from https://methods.cochrane.org/risk-bias-2-faqs#faq-002. This tool provides evaluation in five quality domains: randomization process; deviations from intended interventions; missing outcome data; measurement of the outcome; selection of the reported result. Any differences were resolved by discussion between two researchers (CYM and XHL) until an agreement was reached. Any studies that did not meet an appropriate standard of methodological quality were excluded.

### Quality of the evidence

The Grading of Recommendations, Assessment, Development and Evaluations (GRADE) approach has been used to provide a summary of the evidence’s quality [48]. Using the GRADEprofiler version 3.6 tool, we assess the quality of evidence for the three main outcomes (depression, anxiety, and rumination). The quality of the evidence can be classified into four levels: high, medium, low, and very low. The five indicators that needed to be evaluated are risk of bias, inconsistency, indirectness, imprecision, and publication bias, and they determine whether the quality of evidence should be lower.

### Data Analysis

All searched records were exported to Endnote X9.2. After removing duplicates, two reviewers (CYM and XHL) screened retrieved titles and abstracts and then independently screening the full text for eligibility. Once again, any disagreements were resolved by discussion with the third researcher (YG). When more than one publication was reported by the same author, we only included the newest one with total outcomes data articles. The PICOS approach was used for eligibility criteria (Table 1). Example search strategies details our search procedures [see Supplementary Table 1]. Meta-analysis was performed via RevMan 5.3 software. If the data were not accessible, we would tried contact the author. If the author did not reply, the article was removed. We also summarized the mean, standard deviation, and sample number of the literature in a table [see Supplementary Table 3]. A subgroup analysis is also made to see if there was adequate information [see Supplementary Fig. 4 to 6].

### Data assessment of overall effect size

Since the results of this study were continuous variables, mean change and standard deviation (SD) from baseline to intervention completion were extracted or calculated from the included studies, and weighted mean difference, an effect indicator, was used. The standardized mean difference (SMD) was also used as the summary statistic for the self-report depression, anxiety and rumination, with 95% confidence intervals and two-tailed p-tests conducted for each outcome. We also computed standardized mean differences (SMDs) because different scales were used to measure the same outcomes [49, 50]. A negative SMD was defined as having a benefit to mindfulness yoga compared with the control group. Data conversion is carried out by the RevMan 5.3 software calculator. Cohen’s categories were used to evaluate the magnitude of the overall effect size with (1) small level: SMD = 0.2 to 0.5; (2) medium level: SMD = 0.5 to 0.8; (3) large level: SMD > 0.8 [51]. Separate meta-analyses were conducted for different comparator conditions. When the authors used the same scale to measure the outcome, we chose to count on mean differences (MDs). Subgroup analyses were performed if at least two studies examined the same indicator. The funnel plot has been used to quantify the risk of publication bias, however, due to the small number of included studies, a funnel plot was not included.

### Assessment of heterogeneity

Intervention effect sizes (differences between mindfulness yoga and control groups) for depression, anxiety, and rumination were calculated, along with 95% confidence intervals (Cls) around the estimated effect size. The chi-squared test was performed to generate the Q-statistic, and the I^2^ statistic was calculated for [47]. The random-effects model was used when significant heterogeneity existed (I^2^ > 50%), whereas the fixed-effects model was applied for merging as the result of the absence of significant heterogeneity (I^2^ ≤ 50%). Due to methodological heterogeneity, we perform random-effects analyses on the outcome of depression.

### Sensitivity analysis

A sensitivity test was used as the main source of heterogeneity in our study to indicate the primary determinant of aggregated results. Included studies were excluded one by one to ensure better calculation of the combined effect sizes and their heterogeneity.

### Subgroup analysis

We conducted three subgroup analyses of studies with depression indicators as the primary outcome to investigate the impact of different durations and the types of control group. Considering that the traditional mindfulness-based therapy was eight weeks in length [11], we categorized them at the 8-week time point. Besides, researchers are presently exploring the effects of various intervention durations on individuals, such as six weeks [52], eight weeks [53], and 12 weeks [11]. Thus we also performed a subgroup analysis based on the time points identified in the included studies. In addition, in the section on the control group types, we classified the control groups by whether they were treatment-as-usual (TAU) or other interventions in the literature.

## Results

### Description of studies

**Fig. 1:**
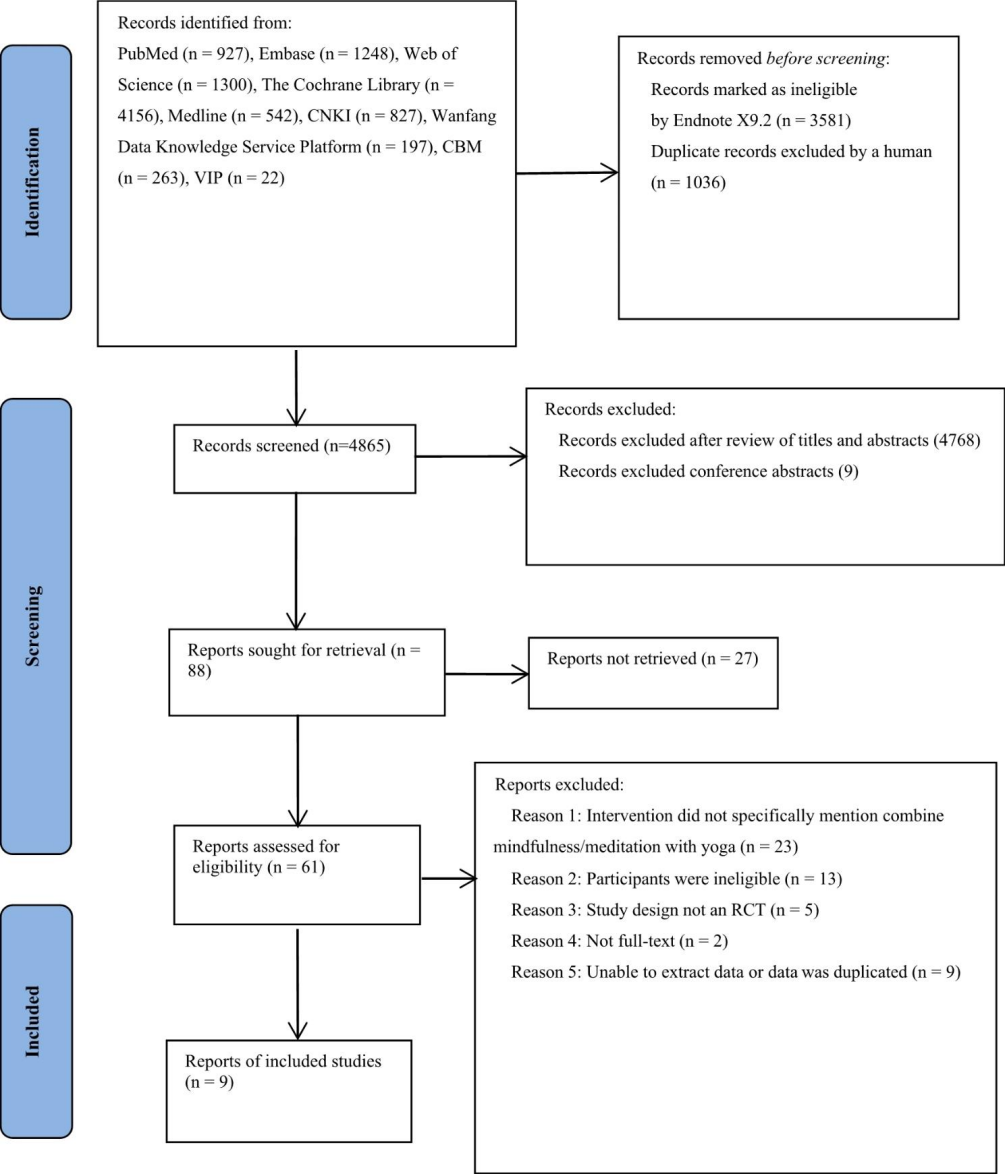
PRISMA flow diagram

A total of 9482 articles were identified in databases. Following the removal of duplicate studies, there were 4865 studies. Then, a further 4777 studies were removed after examination of study titles, abstracts and research type. The remaining 88 studies were potentially relevant. However, 27 studies could not be found or obtained. Therefore 61 studies were retrieved, and the full text assessed. These 61 studies were read independently by two researchers, who made a judgement on them. Any disagreements were resolved through discussion with each other. A further 52 studies were excluded for a variety of reasons (Fig. 1). Therefore, a final total of nine studies were included the review [46]).

### Study characteristics

The nine RCTs in the review were published between 2005 and 2022, detailed elements of these interventions are presented in Supplementary Table 2. Three studies were from the USA [54–56], two was from Germany [57, 58], one was from Netherlands [59], and the other three from India [20, 31, 60]. One study included both major depressive disorder and dysthymia participants [54]. Three studies included participants with current depression [56, 58, 60].

### Participants characteristics

Participants in the identified studies ranged from 18 to 72 years, the total sample number of patients was 581 at baseline and most study participants were female (456/581). Most of the studies provided an explanation of why participants dropped out at different times [31, 54, 56–58]. The main reason for the target population declining to participate in the studies were as follows: individuals said that they were engaged in current yoga practice [56] or not yoga naïve (n = 2 [57]).

### Intervention characteristics

The delivery of the intervention in studies mostly took place within medical institutions, including clinical, hospital, and yoga therapy room. Seven out of nine studies noted that a certified yoga instructor was involved throughout the entire study process, one study was guided by the therapists [58], and only one study provided a DVD for home-based guidance [55]. The duration of the interventions ranged from eight weeks to twelve weeks. The frequency of studies in our review was about one to five times per week, each session lasting between 30 and 120 min.

Most studies shared similar training structure, provided in group form, containing warm-up exercises, sun salutations, stretching exercises, physical strain by a set of physical postures, breathing exercises, and meditation or mindful elements. The intervention were primarily practiced in a supine posture, prone sitting position, and standing [31], with exception of one study which included a special session that was practiced only in a sitting pose, with hands movement at different positions and closed eyes. Besides, mindful practice of the body parts, also called body scan, was regarded as a form of relaxation, an approach practiced from the toes to the top of the head [55, 60]. Mindfulness of body, mind, thoughts, emotions and even eating were also involved within studies [55]. A variety of approaches to yoga were included, two were designed on hatha yoga [54, 56]. Two articles [55, 59] clearly stated that it was mindfulness-based yoga, one [57] was based on body-oriented Ashtanga yoga, one [20] was Sahaj yoga, one was based on classical yoga [58], and the other [31] was not stated. The final study indicated that they provided personalized yoga exercises, which do not fit into any yoga classification [60]. Within our review, some authors described the process and duration of the exercise in great detail [54], whilst others did not.

### Risk-of-bias of randomized controlled trials

According to the Cochrane risk of bias tool (ROB 2.0), four of nine studies had an overall ‘high risk of bias’, and one study raised some concerns [see Supplementary Figs. 1 and 2]. Moreover, included studies unequivocally indicated that participants could not be blinded to treatment assignment, potentially causing them to be aware of the purpose of the study. All included studies reported randomization, with seven out of nine (77.78%) stating that the randomization was computer generated, while in two studies were we unable to know the exact method of randomization it used [20, 58]. Furthermore, random sequence generation and allocation concealment were assessed as inadequate or unclear in included studies. In three of our studies, blinding of participants and personnel was judged high risk [54, 57, 60], whereas in two trials this was deemed ambiguous [20, 31]. Incomplete outcome data were rated as uncertain in one study [31] and as high risk in two others [57, 60], with selective reporting of depression noted in one study [31]. Sample size in included studies ranged from 27 [54] to 88 [59] .

### GRADE analysis of the main outcomes

We (CYM and XHL) used the GRADE score to evaluate the quality of evidence for the three main outcome indicators including depression, anxiety, and rumination. When considering whether or not to downgrade, we examined the outcome based on a combination of considerations, such as heterogeneous outcomes and small sample sizes. The results showed that the evidence quality of the main outcome including depression, anxiety, and rumination indicators were all low [see Supplemental Table 6].

### Overall

The mean and standard deviation of the experimental and control groups were available in nine of the included studies. The overall number of participants included at baseline was 581. Some studies were excluded because of unacceptable statistics. For example, Uebelacker (2018) presents study data with a line graph rather than exact numbers, and we find it hard to abstract it [61]; others, like Vollbehr’s, only provide baseline data [29]. Six participants within Schuver’s [55] study had incomplete assessments after the intervention. We uniformly included post-intervention data indicators to analyze the impact on depression. Since some patients dropped out during the intervention, the total sample of statistics for post-intervention in this review on depression is 568.

#### Depression

**Fig. 2:**
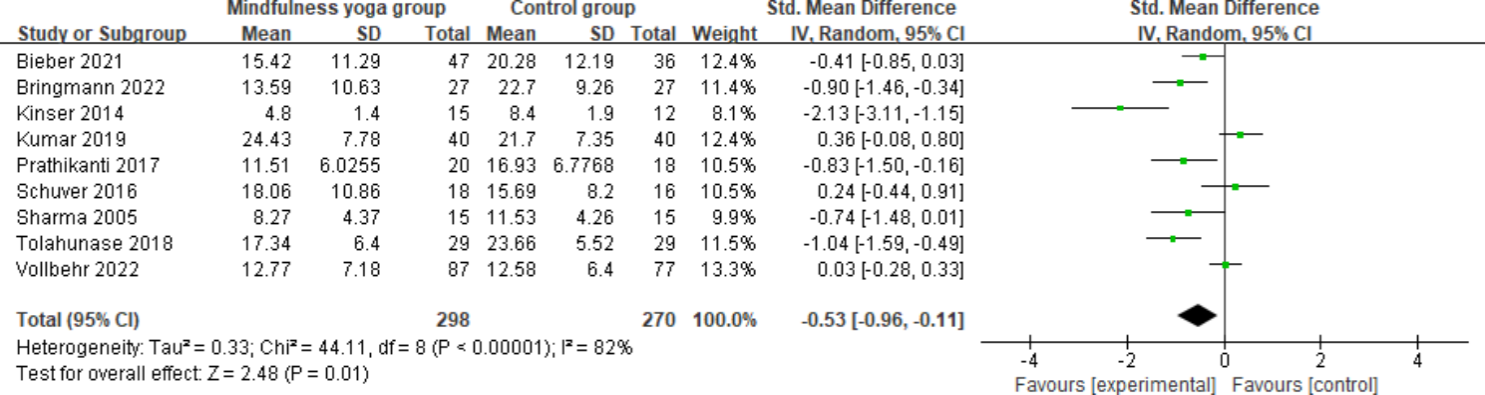
Meta-analysis of mindfulness yoga on depression (post-intervention)

All included studies evaluated the effect of mindfulness yoga on depressive severity measured by the Beck Depression Inventory-II(BDI-II), Montgomery Asberg Depression Rating Scale (MADRS), Hamilton Rating Scale for Depression (HAM-D), Hamilton Depression Rating Scale (HDRS) and Patient Health Questionnaire (PHQ-9). We included all studies in our analysis and found significant statistical heterogeneity (*P* = 0.00001 < 0.01; I^2^ = 82%). Meta-analysis using the random-effects model showed a statistically significant difference in depression levels between patients in the mindfulness yoga group and the control group (SMD = -0.53; 95%CI = -0.96 to -0.11). The results showed that mindfulness yoga might help MDD sufferers minimize their depressed symptoms (Fig. 2 shows the Meta-analysis of mindfulness yoga on post-intervention depression). The significant heterogeneity may be caused by the inconsistent quality of the included literature, according to an analysis of the reasons. This may have resulted from the use of different measurement tools. For example, Schuver and Tolahunase used the BDI scale, Kinser used the PHQ-9 scale, Kumar used the MADRS scale, and Vollbehr used the HDRS scale to measure depressive symptoms.

#### Sensitive analysis of depression

To reduce the impact of heterogeneity, four studies that contributed disproportionately to the pooled effect were identified using sensitivity analyses of all RCTs. Removal of these outliers reduced the degree of heterogeneity between studies from moderate (I^2^ = 82%) to low (0%) and did not materially alter the pooled effect size (medium level) [see Supplementary Fig. 3].

#### Anxiety

**Fig. 3:**
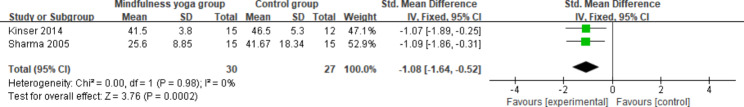
Meta-analysis of mindfulness yoga on anxiety (post-intervention)

Anxiety levels were reported in participants in three studies: Kinser’s using the State-Trait Anxiety Inventory (STAI), Sharma’s using the Hamilton Rating Scale for Anxiety (HAM-D), and Kumar’s using the Hospital Anxiety and Depression Scale (HADS) to measure the effect of mindfulness yoga on alleviating anxiety levels of MDD patients. Kumar et al. reported a statistically significant decrease in anxiety over days 10 and 30. However, as we could not extract the data on anxiety from this study, we decided to omit this study from the analysis of anxiety. Therefore, the combination of only two results showed no statistical heterogeneity between studies (*P* = 0.98; I^2^ = 0%), and a meta-analysis of the fixed-effect model showed that mindfulness yoga could alleviate the anxiety level of MDD patients after intervention (SMD = -1.08; 95%CI = -1.64 to -0.52; *P* = 0.0002). These results are presented in Fig. 3. However, due to the limited literature, mindfulness yoga may have a potential effect on reducing anxiety. As such, more evidence is required to prove the reliability of this conclusion.

#### Rumination

**Fig. 4:**
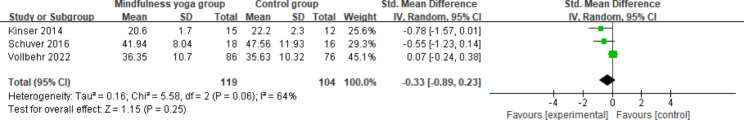
Meta-analysis of mindfulness yoga on rumination (post-intervention)

**Fig. 5:**
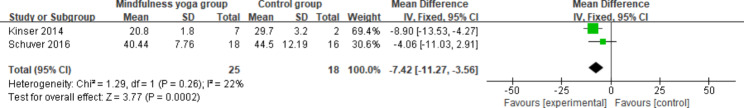
Meta-analysis of mindfulness yoga on rumination (follow-up)

Three studies [54, 55, 59] evaluated the effect of mindfulness yoga on rumination, as measured by the Ruminative Responses Scale and the Perseverative Thinking Scale. Even though the two authors used different acronyms, we found they used the same measurement scale, as seen from their references [54, 55]. Meta-analysis using the fixed effects model did not show a statistically significant difference in rumination between patients in the mindfulness yoga group and the control group after intervention (SMD = -0.33; 95%CI = -0.89 to 0.23; *P* = 0.25) (Fig. 4). There is a large significant statistical heterogeneity after intervention (*P* = 0.06; I^2^ = 64%). We found that rumination in the follow-up period also exists a statistically significant difference (MD = -7.42; 95%CI = -11.27 to -3.56; *P* = 0.0002) (Fig. 5) and with a small significant heterogeneity (*P* = 0.26; I^2^ = 22%). Therefore, there was no sufficient evidence to support that mindfulness yoga could reduce the rumination of MDD patients compared with a control group after intervention. However, the potential benefits of mindfulness yoga on rumination may be shown long after it has taken place.

#### Other outcomes

##### (1) Stress

**Fig. 6:**
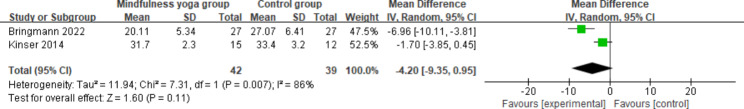
Meta-analysis of mindfulness yoga on stress (post-intervention)

Two studies [54, 58] use the Perceived Stress Scale to measure the effect of mindfulness yoga on stress at 8-week post-intervention. Meta-analysis using the fixed effects model showed there is no statistically significant difference in stress between patients in the mindfulness yoga group and the control group after intervention (MD = -4.20; 95%CI = -9.35 to 0.95; *P* = 0.11), with substantial heterogeneity (*P* = 0.007; I^2^ = 86%) (Fig. 6). Thus, there was no sufficient evidence to support that mindfulness yoga could help reduce stress in MDD patients after the intervention.

##### (2) Quality of life

Two studies [54, 59] reported the effect of mindfulness yoga on health-related quality of life. These were separately measured by the 12-item Short-Form Health Survey (SF-12) and the World Health Organization Quality of Life Questionnaire-BREF. Vollbehr et al. (2022) utilized a bar graph to depict the quality of life of both the mindfulness yoga group and control group. The results showed that while both group experienced an improvement in their quality of life, there was no significant difference between them. Kinser et al. (2014) showed that mindfulness yoga shows effectiveness at the 8-week interventions for the mental component of health-related quality of life (MD = 7.00; 95%CI = 3.91 to 10.09). However, the evidence does not show it has a long-term effect (52 weeks: MD = 3.90; 95%CI = -6.16 to 13.96).

##### (3) Self-efficacy, self-esteem, And self-compassion

Furthermore, another study [56] which reported self-efficacy and self-esteem, noted that neither self-efficacy nor self-esteem significantly differed between the mindfulness yoga group and the control group. In addition, Vollbehr et al. (2022) also found that participants in mindfulness yoga group reported an increase self-compassion and perceived body awareness from baseline to post-intervention.

## Discussion

### Summary of evidence

To the best of our knowledge, this is the first meta-analysis study that try to place a direct focus on the effects of mindfulness yoga on MDD patients. As reported, a comprehensive search strategy was used to ensure identification of the nine relevant RCTs for this review. The purpose of this study was to determine the effectiveness, feasibility and acceptability of mindfulness yoga for MDD patients. The outcome measures we focused on were depression, anxiety, and rumination. Meta-analysis showed that mindfulness yoga had a significant effect on MDD patients on depression. However, there was no significant effect on anxiety, rumination, and other indicators such as stress and quality of life. Thus, based on the limited literatures, the results of this study could only provide initial support for the use of mindfulness yoga for MDD patients.

Although there is a comparable systematic review on the effects of yoga on patients with major depressive disorder [23], our review places more attention to the specific constraints on the types of yoga, and we have attempted to analyze data using quantitative techniques. We discovered that mindfulness yoga might influence depressive symptoms, consistent with previous similar research findings [23, 26, 45]. Moreover, as mindfulness yoga is still at an exploratory stage, the design, duration, and frequency of the interventions may also have a different effect on the outcome. Additionally, as our evidence in the literature is insufficient, the impact of mindfulness yoga on patients with MDD still remains to be explored.

### Strengths of this review

This systematic review and meta-analysis on the effects of mindfulness yoga on MDD has several strengths. Firstly, by rigorously screening the literature, we included only randomized controlled trials, which improved the reliability of our findings by excluding observational and cross-sectional studies. Secondly, our focus on patients with MDD allows us to deliver more targeted and precise interventions in the future. Thirdly, to our knowledge, there is a paucity of research on the intervention of mindfulness yoga for MDD. The study we present is an attempt to explore the effectiveness of an intervention combining elements of mindfulness and yoga in patients with MDD, and thus provides potentially valuable information for patients with MDD in their choice of mind-body movement therapy. Although only measured in a minority of studies, and there was a small evidence base for the long-term effects of mindfulness yoga, particularly in relation to mindfulness level. This may therefore be a promising avenue for future studies in the area.

### Detail content of the review

To eliminate the heterogeneity of the severity of depression, we also performed subgroup analyses by choosing different periods and intervention methods. However, the heterogeneity of effects was noted to be high across all studies.

According to the subgroup analysis of six-week, eight-week, 12-week, and follow-up periods, there is also significant statistical heterogeneity (*P* = 0.0001; I^2^ = 70%). Additionally, according to our meta-analysis, there is no significant difference between the mindfulness yoga group and the control group during these periods using the random-effects model. And it is also shown that heterogeneity between subgroup is within a substantial level (*P* = 0.04; I^2^ = 63.4%) [see Supplementary Fig. 4]. Similarly confirmed by short term (≤ 8-week) and long term (> 8-week) (*P* < 0.0001; I^2^ = 82%, SMD = -0.53; 95%CI = -0.96 to -0.11) [see Supplementary Fig. 5]. Our study only found an improvement in depressive symptoms in MDD patients in eight-weeks compared to six-week, 12-week and follow-up period, but there remained a moderate heterogeneity (*P* = 0.11; I^2^ = 50%) [see Supplementary Fig. 4]. One study also reported slight effectiveness of mindfulness yoga during a 2.5-month period, which is similar to the conclusion we reached [62]. There remains a lack of evidence to support that mindfulness and yoga can maintain their effectiveness in the longer term. One interpretation of Prathikanti’s study is that there may be delayed effectiveness over time. It has been speculated that more frequent sessions are connected to reducing anxiety symptoms, although we found no difference at different periods [27]. We conclude that more practice would appear more efficacious [26].

As for subgroups of different interventions, there was no difference when comparing the mindfulness yoga group with other treatments group such as attention control (SMD = -0.86; 95%CI = -2.09 to 0.37), and the treatment-as-usual group (SMD = -0.41; 95%CI = -0.85 to 0.03) [see Supplementary Fig. 6]. These conclusions are consistent with Cramer et al. findings [23]. Consequently, the results of both subgroups did not demonstrate that mindfulness yoga intervention can significantly reduce depression. Nevertheless, this finding may be due to the influence of many confounding factors.

### Limitations and future prospects

Despite the strengths of our study, some potential limitations should be noted. Firstly, there is heterogeneity resulting from the different measuring tools and potential bias. Second, our study included yoga incorporating elements of mindfulness or meditation, which may have a degree of heterogeneity that weakened the ability of meta-analyses to show clear and consistent effects. As a result, we may not be able to fully explain in a specific way how to distinguish between the manifestations of traditional yoga and mindfulness yoga. Thirdly, there exist significant baseline differences in rumination. With limited studies, we could only find mindfulness yoga to have a potential effect on reducing levels of rumination. Only two studies reported follow-up results: one at one year [54] and the other at only one month [55]. Another potential limitation relates to our inability to extract some of the data from included randomized controlled articles, and due to the small number of included studies, the results should be treated with caution. Besides, there is insufficient evidence to ascertain whether longer-term or shorter-term mindfulness training is better. To find an effective, high-quality practice cutoff time, further research should analyze intervention terms. It should also compare different period types in a larger sample with a more rigorous reporting methodology. And biological indicators, such as the interleukin (IL) and C-reactive protein (CRP) biomarkers, are required to be used to provide more reliable evidence for this intervention. Finally, if conditions allow, it is also suggested to compare the effects of mindfulness yoga and traditional yoga on those with MDD, respectively.

## Conclusion

Mindfulness yoga might be a promising non-pharmacological intervention for MDD patients. However, due to the small amount of literature on this study and the various implementation of the current intervention approach, a more rigorous design of mindfulness yoga intervention, a large sample size, and high-quality studies are needed to evaluate the efficacy of mindfulness yoga further and validate our findings.

### Electronic supplementary material

Below is the link to the electronic supplementary material.

Supplementary Material 1

## Data Availability

The data supporting the conclusions of this review are included within the article tables, figures and supplementary information.

## List of abbreviations

RCTs: Randomized Controlled Trials
MDD: Major Depressive Disorder
PRISMA: Preferred Reporting Items for Systematic Reviews and Meta-analyses
GRADE: The Grading of Recommendations, Assessment, Development and Evaluations
SMD: Standard Mean Differences
MD: Mean Differences
CI: Confidence Interval
