# Supplementary material for: The effectiveness of mindfulness yoga on patients with major depressive disorder: a systematic review and meta-analysis of randomized controlled trials
Source: BMC Complement Med Ther. 2023 Sep 8;23:313. doi: 10.1186/s12906-023-04141-2 (PMC10492419; doi:10.1186/s12906-023-04141-2)
Supplement: Supplementary file 1 — Supplementary Material 1 [file 12906_2023_4141_MOESM1_ESM.docx]

**Supplementary material**

The effectiveness of mindfulness yoga on patients with major depressive disorder: a systematic review and meta-analysis of randomized controlled trials

| Supplementary Contents | Contents | Page |
| --- | --- | --- |
| Supplementary Figure 1 | Cochrane risk of bias graph: review authors’ judgments about each risk of bias item presented as percentages across all included studies. | 2 |
| Supplementary Figure 2 | Cochrane risk of bias summary: review authors’ judgment about each risk of bias item for each included study. | 3 |
| Supplementary Figure 3 | Meta-analysis of mindfulness yoga on depression (sensitivity analyses) | 4 |
| Supplementary Figure 4 | Subgroup of depression (division by specific time period) | 5 |
| Supplementary Figure 5 | Subgroup of depression (division by short-term or long-term period) | 6 |
| Supplementary Figure 6 | Subgroup of depression (control group with different interventions) | 7 |
| Supplementary Table 1 | Retrieval search strategy sample of PubMed | 8 |
| Supplementary Table 2 | Data extraction of the included studies | 9-13 |
| Supplementary Table 3 | Mean, standard deviation, and sample size used in the meta-analysis | 14-17 |
| Supplementary Table 4 | Summary of the included studies’ strengths and weaknesses | 18-20 |
| Supplementary Table 5 | PRISMA checklist | 21-25 |
| Supplementary Table 6 | GRADE analysis of the primary and secondary outcomes | 26 |

**Supplementary Figure 1.** Cochrane risk of bias graph: review authors’ judgments about each risk of bias item presented as percentages across all included studies.

|  | | Randomization process | | Deviations from intended interventions | | Missing outcome data | | Measurement of the outcome | | Selection of the reported result | | Overall | |  | |  | |  | |  | |  | |
| --- | --- | --- | --- | --- | --- | --- | --- | --- | --- | --- | --- | --- | --- | --- | --- | --- | --- | --- | --- | --- | --- | --- | --- |
| Bieber2021 | | 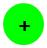 | | 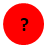 | | 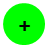 | | 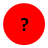 | | 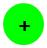 | | 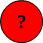 | |  | |  | |  | |  | |  | |
| Kinser2014 | | 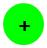 | | 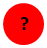 | | 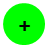 | | 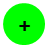 | | 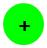 | | 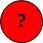 | |  | |  | |  | |  | |  | |
| Kumar2019 | | 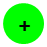 | | 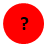 | | 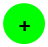 | | 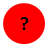 | | 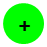 | | 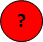 | |  | |  | |  | |  | |  | |
| Prathikanti2017 | | 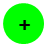 | | 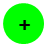 | | 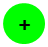 | | 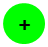 | | 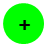 | | 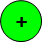 | |  | |  | |  | |  | |  | |
| Schuver2016 | | 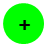 | | 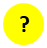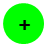 | | 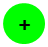 | | 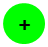 | | 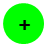 | | 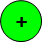 | |  | |  | |  | |  | |  | |
| Sharma2005 | | 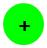 | |  | | 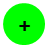 | | 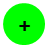 | | 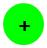 | | 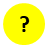 | |  | |  | |  | |  | |  | |
| Tolahunase2018 | | 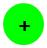 | | 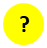 | | 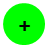 | | 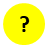 | | 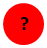 | | 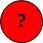 | |  | |  | |  | |  | |  | |
| Bringmann2022 | | 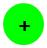 | | 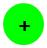 | | 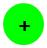 | | 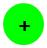 | | 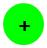 | |  | |  | |  | |  | |  | |  | |
| Vollbehr2022 | | 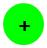 | | 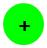 | | 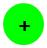 | | 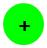 | | 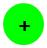 | |  | |  | |  | |  | |  | |  | |
| **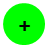** | | Low risk | | | | | | 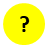 | | Some concerns | | | | | | 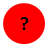 | | High risk | | | | | |

**Supplementary Figure 2.** Cochrane risk of bias summary: review authors’ judgments about each risk of bias item for each included study.


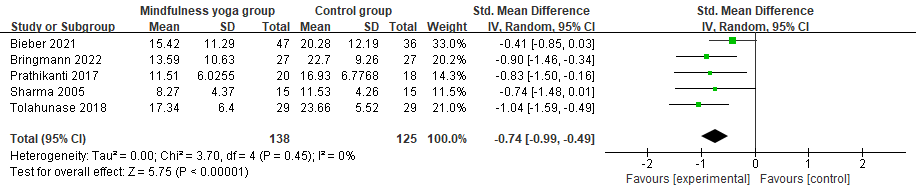


**Supplementary Figure 3.** Meta-analysis of mindfulness yoga on depression (sensitivity analyses)


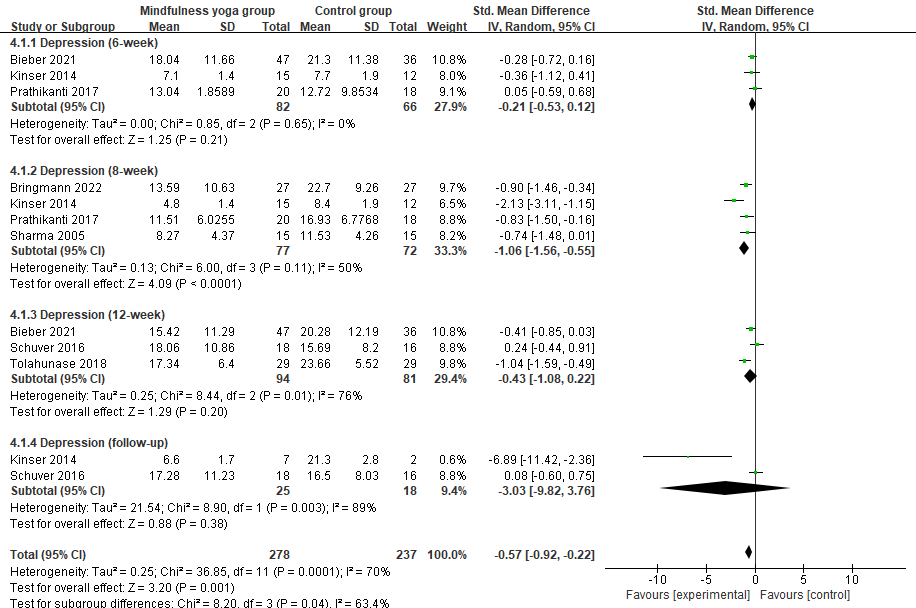


**Supplementary Figure 4.** Subgroup of depression (division by specific time period)

**
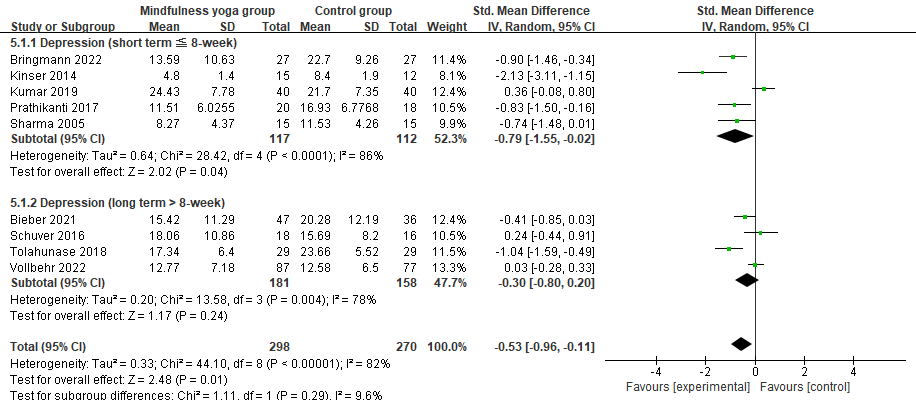
**

**Supplementary Figure 5.** Subgroup of depression (division by short-term or long-term period)


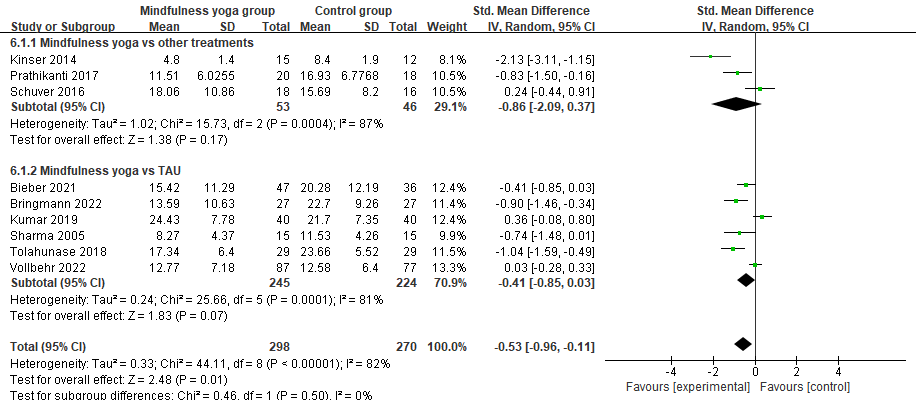


**Supplementary Figure 6.** Subgroup of depression (control group with different interventions)

**Supplementary Table 1.** Retrieval search strategy sample of PubMed

|  | PUBMED |
| --- | --- |
| #1 | (((depressive disorder[MeSH Terms]) OR (depression[MeSH Terms])) OR (depressi*[Title/Abstract])) |
| #2 | (yoga [MeSH Terms]) OR (yoga[Title/Abstract]) |
| #3 | ((mindfulness[MeSH Terms]) OR (meditation[MeSH Terms]) OR (mindful*[Title/Abstract]) |
| #4 | #2 OR #3 |
| #5 | #4 AND #1 |
| #6 | Randomized Controlled Trial |
| #7 | English |
| #8 | #5 AND #6 AND #7 |
| Total | 927 |

**Total search formula: (((depressive disorder[MeSH Terms]) OR (depression[MeSH Terms])) OR (depressi*[Title/Abstract])) AND (((yoga[MeSH Terms]) OR (yoga[Title/Abstract])) OR (((mindfulness[MeSH Terms]) OR (meditation[MeSH Terms])) OR (mindful*[Title/Abstract]))) Filters: Randomized Controlled Trial, English**

**Supplementary Table 2.** Data extraction of the included studies

| **The first author/Year/**  **Country** | **Participants’ age: mean(SD)** | **Allocation** | **Intervention group/ duration/time/**  **frequency** | **Content of Intervention** | **Comparison group** | **Measurement** | **Outcome** |
| --- | --- | --- | --- | --- | --- | --- | --- |
| Nina K. Vollbehr/2022/Netherlands | IG:25.20±4.90  CG:24.90±4.36 | IG: 88  CG: 83 | MYI(Mindful yoga)/9 weeks/1.5h/once a week | Included yoga postures, breathing practices, meditation practices of traditional hatha yoga | TAU | ①HDRS  ②PTQ | PO: depressive severity  SO: rumination, well-being, quality of life, mindfulness, etc. |
| Holger C. Bringmann/2022/Germany | IG: 49.1±11.1  CG: 45.0±11.1 | IG: 27  CG: 27 | MBLM(Meditation Based Lifestyle Modification)/8 weeks/180minutes/once a week | Included the eightfold path of classical yoga(ethical living, healthy lifestyle, and mantra meditation), breathing exercises, mantra meditation | Multidisciplinary psychiatric outpatient care (TAU): receiving pharmacotherapy, psychotherapy, and accessory therapies, etc. | ①BDI-Ⅱ  ②PSS-10 | PO: depressive severity  SO: the perception of stress. |
| Bieber, M./  2021/Germany | Total: 49.65±9.83  IG: 48.38±10.21  CG: 51.31±9.19 | IG: 47  CG: 36 | Yoga-exercise/10-12 patients group/12weeks/90minutes/three times per week | Consist of sequences of body-oriented Ashtanga-Yoga: warm-up, sun salutations, stretching exercises, dynamic and active yoga practice, cool-down sun salutations and stretching, breath-controlling and meditation-elements | Waiting-list control group  (Received a TAU, consisting of antidepressant medication and psychotherapy.) | ①BDI-Ⅱ  ②MADRS  ③PANAS | PO: depressive syndrome  SO: individual positive and negative affectivity, remission rates |
| Kumar, S./  2019/India | Total: 38.19(11.73)  IG: 36.90(10.17)  CG: 39.48(13.29) | IG: 40  CG: 40 | Yoga/minimum 20 supervised yoga sessions/45minutes/  five sessions per week | Included gentle stretching, meditation, simple warm-up and breath body movement coordination practices, static stretching postures, breathing techniques, and relaxation, alternate nostril breathing, slow and deep breathing.  Each session ended with relaxation for 10 min in the supine shava asana with mindful relaxation of the body parts from toes to top of the head | Standard therapy/TAU (antidepressants and psychological intervention) | ①MADRS  ②HADS  ③CGI | PO: depression, anxiety  SO: severity of illness and clinical improvement |
| Madhuri R. Tolahunase/2018/India | IG:36.94(8.94)  CG:39.10(9.26) | IG:29  CG:29 | YMLI with routine drug treatment/12weeks/120minutes/five days per week | Included prayer, loosening exercises, sun salutation, asanas (physical postures), relaxation, aumkar recitation, shanti mantra, pranayama (breathing exercises), and dhyana (meditation), interactive session/self-directed learning | TAU (Routine drug treatment only) | BDI-Ⅱ scale | PO: depression severity  SO: Systemic biomarkers of neuroplasticity： BDNF; mind-body communication biomarkers; biomarkers of cellular health-oxidative stress |
| Prathikanti, Sudha/  2017/USA | Full Sample: 43.4(14.8)  IG: 43.1(15.2)  CG: 43.8(14.7) | IG: 20  CG: 18 | Hatha yoga intervention  /8 weeks/90 minutes/  twice weekly | Comprised of classical yoga breathing techniques, mindful body postures/movement, deep relaxation pose | Attention control group (a series of community lectures) | ①BDI-Ⅱ  ②GSES  ③RSES | PO: depression symptoms  SO: self-efficacy,  self-esteem |
| Schuver, K. J./  2016/USA | Total: 42.68(4.95)  IG: 45.55(12.30)  CG: 39.8(11.23) | IG: 20  CG: 20  (IG: 18, CG: 16 after intervention) | Mindfulness-based yoga/12weeks/60-75minutes/twice per week | 12-week mindfulness-based yoga condition was guided by a gentle yoga DVD that included postures, breathing exercises, and relaxation (meditation) | The 12-week walking (Included twice-weekly home practice with a 65-min walking DVD) | ①BDI  ②RSS | PO: depressive symptoms  SO: ruminating thoughts |
| Kinser, P. A./2014/USA | Overall: 43.3(15.6)  IG: 40.9(15.8)  CG: 46.2(15.4)  52Weeks follow-up：  IG: 38.3(13.3)  CG: 41.0(14.1) | IG: 15  CG: 12  52 Weeks follow-up:  IG: 7  CG: 2 | Yoga intervention group/8 weeks/75 minutes/weekly | Included 75minutes local yoga studios, gentle hatha yoga movements, breathing practices, physical movements, meditative self-inquiry/relaxation practices | Attention-control group (The 75-minute weekly sessions involved lectures, videos, and discussions about alcohol safety, sleep, nutrition, heart health, bone health, depression, anxiety, and stress) | ①PHQ-9  ②PSS-10  ③RRS  ④STAI  ⑤SF-12 | PO: ①depression severity; ②stress; ③rumination; ④anxiety; ⑤health related quality of life |
| V. K. Sharma/  2005/India | IG: 31.87±8,78  CG: 31.67±8.46 | IG: 15  CG: 15 | Sahaj Yoga and conventional antidepressants/8 weeks/30 minutes/three times per week | Included Sahaj yoga, sit and silent meditation with the eyes closed | Only receiving conventional anti-depressants | ①HAM-D  ②HAM-A | PO: depression severity  SO: anxiety |

**Abbreviation: IG, Intervention group; CG, Comparison group; TAU, Treatment-as-usual; YMLI, Yoga- and meditation-based lifestyle intervention; PO, Primary outcome; SO, Secondary outcome; BDI-Ⅱ, Beck Depression Inventory-Ⅱ; MADRS, Montgomery Asberg Depression Rating Scale; HADS, Hospital Anxiety and Depression Scale; HAM-D, Hamilton Rating Scale for Depression; HAM-A, Hamilton Rating Scale for Anxiety; QIDS, Quick Inventory of Depression Symptomatology – Clinician Rating; PHQ-9, Patient Health Questionnaire, nine items; STAI, the State Trait Anxiety Inventory; PSS-10, Perceived Stress Scale-10; SF-12, 12-item Short Form Health Survey; CGI, The Clinical Global Impression scale; RSES, Rosenberg Self-esteem Scale; RSS, The Ruminative Responses Scale; RRS, Ruminative Responses Scale; GSES, General self-efficacy scale; PANAS, The Positive and Negative Affect Scale; HDRS, the Hamilton Depression Rating Scale; PTQ, Perseverative Thinking Scale.**

**Supplementary Table 3.** Mean, standard deviation, and sample size used in the meta-analysis

| **Mindfulness yoga group** | | | | | | | | | | | |  | **Control group** | | | | | | | | | |  |
| --- | --- | --- | --- | --- | --- | --- | --- | --- | --- | --- | --- | --- | --- | --- | --- | --- | --- | --- | --- | --- | --- | --- | --- |
| **Study** | | | **Measurement** | **Baseline mean (SD)** | **2-week mean (SD)** | **4-week mean (SD)** | **6-week mean (SD)** | **8-week mean (SD)** | **9-week mean (SD)** | **12-week mean (SD)** | **Follow-up** | **N** |  | **Baseline mean (SD)** | **2-week mean (SD)** | **4-week mean (SD)** | | **6-week mean (SD)** | **8-week mean (SD)** | **9-week mean (SD)** | **12-week mean (SD)** | **Follow-up** | **N** |
| Vollbehr, 2022 | | HDRS | | 19.05 (5.81)  (n=87) | N/A | N/A | N/A | N/A | 12.77 (7.18)  (n=87) | N/A | 6m:10.77 (7.69);  12m:7.25 (7.03) | 88 |  | 18.07 (6.06)  (n=83) | N/A | N/A | | N/A | N/A | 12.58 (6.40)  (n=77) | N/A | 6m:9.29 (7.08);  12m:8.06 (7.49) | 83 |
|  |  | PTQ | | 42.14 (7.85)  (n=87) | N/A | N/A | N/A | N/A | 36.35 (10.7)  (n=86) | N/A | N/A |  |  | 41.44 (8.60)  (n=81) | N/A | N/A | | N/A | N/A | 35.63 (10.32)  (n=76) | N/A | N/A |  |
| Bringmann, 2022 | BDI-II | | 26.74 (9.46) | N/A | 16.81 (10.65) | N/A | 13.59 (10.63) | N/A | N/A | N/A | 27 |  | TAU: 26.4 (8.12) | N/A | 24.33 (7.78) | | N/A | 22.70 (9.26) | N/A | N/A | N/A | 27 |  |
|  | PSS-10 | | 28.04 (4.60) | N/A | N/A | N/A | 20.11 (5.34) | N/A | N/A | N/A |  |  | TAU: 28.26 (4.93) | N/A | N/A | | N/A | 27.07 (6.41) | N/A | N/A | N/A |  |  |
| Bieber, 2021 | BDI-II | | 23.19 (11.36) | N/A | N/A | 18.04 (11.66) | N/A |  | 15.42 (11.29) | N/A | 47 |  | 26.08 (13.07) | N/A | N/A | | 21.30 (11.38) | N/A |  | 20.28 (12.19) | N/A | 36 |  |
|  | MADRS | | 24.26  (8.09) | N/A | N/A | 18.45 (10.28) | N/A |  | 15.25 (7.39) | N/A |  |  | 23.18  (10.29) | N/A | N/A | | 20.05 (9.03) | N/A |  | 18.3 (7.61) | N/A |  |  |
| Prathikanti, 2017 | BDI-II | | 20.98 (5.4058) | 15.15 (6.3673) | 14.35 (8.3453) | 13.04 (1.8589) | 11.51 (6.0255) |  | N/A | N/A | 20 |  | 19.92 (5.3490) | 13.06 (8.3453) | 14.66 (7.8425) | | 12.72 (9.8534) | 16.93 (6.7768) |  | N/A | N/A | 18 |  |
|  | GSES | | 26.87 (3.09) | N/A | N/A | N/A | 29.0 (3.89) |  | N/A | N/A | 15 |  | 28.5 (4.33) | N/A | N/A | | N/A | 30.0 (2.67) |  | N/A | N/A | 10 |  |
|  | RSES | | 14.6 (3.48) | N/A | N/A | N/A | 17.47 (3.87) |  | N/A | N/A | 15 |  | 16.0 (3.77) | N/A | N/A | | N/A | 16.2 (3.88) |  | N/A | N/A | 10 |  |
| Tolahunase, 2018 | BDI-II | | 23.17 (4.31) | N/A | N/A | N/A | N/A |  | 17.34 (6.4) | N/A | 29 |  | 22.21 (4.39) | N/A | N/A | | N/A | N/A |  | 23.66 (5.52) | N/A | 29 |  |
| Schuver, 2016 | BDI | | 26.20 (6.60) | N/A | N/A | N/A | N/A |  | 18.06 (10.86) | 1m:17.28 (11.23) | 18 |  | 26.50 (5.50) | N/A | N/A | | N/A | N/A |  | 15.69 (8.20) | 16.50 (8.03) | 16 |  |
|  | RRS | | 60.20 (10.38) | N/A | N/A | N/A | N/A |  | 41.94 (8.04) | 40.44 (7.76) |  |  | 58.45 (9.61) | N/A | N/A | | N/A | N/A |  | 47.56 (11.93) | 44.50 (12.19) |  |  |
| Kinser, 2014 | PHQ-9 | | 14.9 (1.3) | 11.6 (1.4) | 7.9 (1.4) | 7.1 (1.4) | 4.8 (1.4) |  | N/A | 52w: 6.6 (1.7) | 15  (52w:7) |  | 16.4 (1.5) | 14.4 (1.8) | 8.9 (1.9) | | 7.7(1.9) | 8.4 (1.9) |  | N/A | 52w: 21.3 (2.8) | 12  (52w: 2) |  |
|  | PSS-10 | | 38.5 (2.1) | N/A | 35.6 (2.2) | N/A | 31.7 (2.3) |  | N/A | 52w: 37.3 (2.9) |  |  | 38 (2.3) | N/A | 37.4 (3.2) | | N/A | 33.4 (3.2) |  | N/A | 52w: 38.5 (5.4) |  |  |
|  | RRS | | 27.4 (1.6) | N/A | 24.1 (1.7) | N/A | 20.6 (1.7) |  | N/A | 52w: 20.8 (1.8) |  |  | 24.9 (1.8) | N/A | 23.7 (2.2) | | N/A | 22.2 (2.3) |  | N/A | 52w: 29.7 (3.2) |  |  |
|  | STAI | | 52.5 (3.5) | N/A | 47.1 (3.7) | N/A | 41.5 (3.8) |  | N/A | 38.5 (4.7) |  |  | 55.1 (3.9) | N/A | 48.9 (5.3) | | N/A | 46.5 (5.3) |  | N/A | 56.5 (8.5) |  |  |
|  | Mental Component of HRQoL (SF-12) | | 24.5 (2.8) | N/A | 36.8 (3) | N/A | 45.9 (3.1) |  | N/A | 36.8 (4.2) |  |  | 25.8 (3.2) | N/A | 33.3 (4.3) | | N/A | 38.9 (4.7) |  | N/A | 32.9 (6.9) |  |  |
| Sharma, 2005 | HAM-D | | 21.27 (4.35) | N/A | N/A | N/A | 8.27 (4.37) |  | N/A | N/A | 15 |  | 19.47 (3.98) | N/A | N/A | | N/A | 11.53(4.26) |  | N/A | N/A | 15 |  |
|  | HAM-A | | 58.00(14.60) | N/A | N/A | N/A | 25.60(8.85) |  | N/A | N/A |  |  | 59.33(17.52) | N/A | N/A | | N/A | 41.67(18.34) |  | N/A | N/A |  |  |
| Kumar, 2019 | MADRS | | Baseline-30d: 24.43(7.78) | | | | |  | N/A | N/A | 40 |  | Baseline-30d: 21.70(7.35) | | | | | |  | N/A | N/A | 40 |  |
|  | HADS | | Difficult to extract and of little significance | | | | |  | N/A | N/A |  |  | N/A | N/A | N/A | N/A | |  | N/A | N/A | N/A |  |  |
|  | CGI | | Difficult to extract and of little significance | | | | |  | N/A | N/A |  |  | N/A | N/A | N/A | N/A | |  | N/A | N/A | N/A |  |  |

**Abbreviation: m, month(s); d,day(s); w, week(s); TAU, treatment as usual; N/A, Not applicable;**

**Supplementary Table 4.** Summary of the included studies’ strengths and weaknesses

| Included studies (first author) | Strengths | Weaknesses | Future |
| --- | --- | --- | --- |
| Vollbehr | 1. A relatively larger sample.  2. The study design is reliable.  3. After a comparatively lengthy one-year follow-up period, it is possible to draw longer-term conclusions. | 1. The sample is limited to young women suffering from clinical depression.  2. The sample size limits the generalizability of the findings. | 1. Future research should compare the similarities and differences of mindful yoga intervention across gender, age, and ethnicity. |
| Bringmann | The first trial to evaluate the efficacy of the MBLM (Meditation Based Lifestyle Modification, an 8-week mind-body intervention) program in patients with depression. | 1. The study was specifically designed for depression patients interested in yoga or meditation. The results cannot therefore be generalized to depressed outpatients.  2. The trial was conducted in only one center by the developers of the MBLM program, which may have inflated effect sizes due to allegiance effects. | 1. Future controlled clinical research, including qualitative ones, needs to be high-quality.  2. The ethical and philosophical aspects of underlying traditions like yoga or other meditative practices are underrepresented in mind–body interventions research and should be investigated to test their therapeutic utility.  3. Theory-driven basic research is needed to completely advance research. |
| Bieber | 1. Patients with regular yoga training experienced a slightly greater improvement in depressive symptoms and mood).  2. The effect sizes showed a high effect for the yoga group. | 1. A lack of a fully randomized study design.  2. A lack of power to secure the detection of the identified effects.  3. High dropout level (statistical performance). | 1. Valid representations of mood changes require more frequent measurements.  2. Ashtanga Yoga needs to be compared with other mindfulness-based styles. |
| Prathikanti | Feasibility and effectiveness data to support a full-scale RCT of hatha yoga as a single treatment for mild-to-moderate major depression in a sample of non-hospitalized, metropolitan U.S. adults. | 1. A pilot study with small sample size.  2. Hard to figure out what the "minimum effective dose" of hatha yoga is.  3. Insufficient evidence to recommend yoga as a first-line treatment for depression. | A larger scale RCTs with hatha yoga. |
| Tolahunase | This was the first RCT of yoga studying its impact on cellular health and mind-body communicative factors on neuroplasticity and depression severity in cases with MDD patients. | 1. Small sample size.  2. Not reporting longer-term follow-up effects. | Using an integrated health strategy to prevent and treat MDD patients. |
| Schuver | 1. Use well-validated, reliable measurement tools  2. Focus on mindfulness training for the intervention (yoga) condition.  3. Individual was blind to the condition assignment.  4. Random allocation procedure was used.  5. Had high adherence to the study protocol and low attrition numbers. | 1. Major participants were Caucasian, educated, employed, and of mid-high socioeconomic status.  2. A small sample size.  3. The measurement were self-report.  4. Participants were aware of the purpose of the study (social desirability bias). | 1. Include a wide range of sample sizes, interventions that last longer, and long-term follow-ups.  2. Include biomarker measures (like cortisol) of depression and anxiety symptoms and objective measurements of physical activity. |
| Kinser | Evaluate long-term outcomes. | Small sample size (volunteer bias). | Large-scale studies are needed.  (e.g., create an intervention protocol) |
| Sharma | Stable response. | The mechanism of Sahaj Yoga for reducing depression cannot be deciphered. | 1. Longer periods of Sahaj Yoga practice.  2. Comparative studies with other forms of Yoga are also needed. |
| Kumar | The results may be considered more reliable due to the study ensured increased adherence to yoga sessions. | 1. The therapist spent more time with the yoga group than with other groups. And the study’s design did not take that into account.  2. The yoga group lacked a precise procedure, resulting in a lack of homogeneity in the treatment received.  3. Lack of blinding. | A large sample, longer follow-up study. |

**Supplementary Table 5.** PRISMA checklist

| **Section and Topic** | **Item #** | **Checklist item** | **Location where item is reported** |
| --- | --- | --- | --- |
| **TITLE** | | |  |
| Title | 1 | Identify the report as a systematic review. | P1 |
| **ABSTRACT** | | |  |
| Abstract | 2 | See the PRISMA 2020 for Abstracts checklist. | P1-P2 |
| **INTRODUCTION** | | |  |
| Rationale | 3 | Describe the rationale for the review in the context of existing knowledge. | P2-P6 |
| Objectives | 4 | Provide an explicit statement of the objective(s) or question(s) the review addresses. | P6 |
| **METHODS** | | |  |
| Eligibility criteria | 5 | Specify the inclusion and exclusion criteria for the review and how studies were grouped for the syntheses. | Table 1 |
| Information sources | 6 | Specify all databases, registers, websites, organisations, reference lists and other sources searched or consulted to identify studies. Specify the date when each source was last searched or consulted. | P6-P7 |
| Search strategy | 7 | Present the full search strategies for all databases, registers and websites, including any filters and limits used. | P7; Fig. 1 |
| Selection process | 8 | Specify the methods used to decide whether a study met the inclusion criteria of the review, including how many reviewers screened each record and each report retrieved, whether they worked independently, and if applicable, details of automation tools used in the process. | P7-P8 |
| Data collection process | 9 | Specify the methods used to collect data from reports, including how many reviewers collected data from each report, whether they worked independently, any processes for obtaining or confirming data from study investigators, and if applicable, details of automation tools used in the process. | P7-P8 |
| Data items | 10a | List and define all outcomes for which data were sought. Specify whether all results that were compatible with each outcome domain in each study were sought (e.g. for all measures, time points, analyses), and if not, the methods used to decide which results to collect. | P6-P11;  Supplementary Table 2 and 3 |
|  | 10b | List and define all other variables for which data were sought (e.g. participant and intervention characteristics, funding sources). Describe any assumptions made about any missing or unclear information. | Supplementary Table 2 and 3 |
| Study risk of bias assessment | 11 | Specify the methods used to assess risk of bias in the included studies, including details of the tool(s) used, how many reviewers assessed each study and whether they worked independently, and if applicable, details of automation tools used in the process. | P8 |
| Effect measures | 12 | Specify for each outcome the effect measure(s) (e.g. risk ratio, mean difference) used in the synthesis or presentation of results. | P9-P10 |
| Synthesis methods | 13a | Describe the processes used to decide which studies were eligible for each synthesis (e.g. tabulating the study intervention characteristics and comparing against the planned groups for each synthesis (item #5)). | Supplementary Table 2 |
|  | 13b | Describe any methods required to prepare the data for presentation or synthesis, such as handling of missing summary statistics, or data conversions. | P9 |
|  | 13c | Describe any methods used to tabulate or visually display results of individual studies and syntheses. | P8 |
|  | 13d | Describe any methods used to synthesize results and provide a rationale for the choice(s). If meta-analysis was performed, describe the model(s), method(s) to identify the presence and extent of statistical heterogeneity, and software package(s) used. | P8-P11 |
|  | 13e | Describe any methods used to explore possible causes of heterogeneity among study results (e.g. subgroup analysis, meta-regression). | P10-P11 |
|  | 13f | Describe any sensitivity analyses conducted to assess robustness of the synthesized results. | P10-P11 |
| Reporting bias assessment | 14 | Describe any methods used to assess risk of bias due to missing results in a synthesis (arising from reporting biases). | / |
| Certainty assessment | 15 | Describe any methods used to assess certainty (or confidence) in the body of evidence for an outcome. | P9 |
| **RESULTS** | | |  |
| Study selection | 16a | Describe the results of the search and selection process, from the number of records identified in the search to the number of studies included in the review, ideally using a flow diagram. | Fig. 1 |
|  | 16b | Cite studies that might appear to meet the inclusion criteria, but which were excluded, and explain why they were excluded. | P11-P13; Fig. 1 |
| Study characteristics | 17 | Cite each included study and present its characteristics. | P11-P13; |
| Risk of bias in studies | 18 | Present assessments of risk of bias for each included study. | P13-P14; |
| Results of individual studies | 19 | For all outcomes, present, for each study: (a) summary statistics for each group (where appropriate) and (b) an effect estimate and its precision (e.g. confidence/credible interval), ideally using structured tables or plots. | Fig. 2 to Fig. 6 |
| Results of syntheses | 20a | For each synthesis, briefly summaries the characteristics and risk of bias among contributing studies. | P12-P15; Supplementary Figure 1 and 2 |
|  | 20b | Present results of all statistical syntheses conducted. If meta-analysis was done, present for each the summary estimate and its precision (e.g. confidence/credible interval) and measures of statistical heterogeneity. If comparing groups, describe the direction of the effect. | P15-P18 |
|  | 20c | Present results of all investigations of possible causes of heterogeneity among study results. | P15-P18 |
|  | 20d | Present results of all sensitivity analyses conducted to assess the robustness of the synthesized results. | P15 |
| Reporting biases | 21 | Present assessments of risk of bias due to missing results (arising from reporting biases) for each synthesis assessed. | Supplementary Figure 1 and 2 |
| Certainty of evidence | 22 | Present assessments of certainty (or confidence) in the body of evidence for each outcome assessed. | Supplementary Table 6 |
| **DISCUSSION** | | |  |
| Discussion | 23a | Provide a general interpretation of the results in the context of other evidence. | P18 |
|  | 23b | Discuss any limitations of the evidence included in the review. | P21 |
|  | 23c | Discuss any limitations of the review processes used. | P20-P21 |
|  | 23d | Discuss implications of the results for practice, policy, and future research. | P22-P23 |
| **OTHER INFORMATION** | | |  |
| Registration and protocol | 24a | Provide registration information for the review, including register name and registration number, or state that the review was not registered. | P6 |
|  | 24b | Indicate where the review protocol can be accessed, or state that a protocol was not prepared. | P6 |
|  | 24c | Describe and explain any amendments to information provided at registration or in the protocol. | P6 |
| Support | 25 | Describe sources of financial or non-financial support for the review, and the role of the funders or sponsors in the review. | P25 |
| Competing interests | 26 | Declare any competing interests of review authors. | P25 |
| Availability of data, code and other materials | 27 | Report which of the following are publicly available and where they can be found: template data collection forms; data extracted from included studies; data used for all analyses; analytic code; any other materials used in the review. | P23 |

*From:*  Page MJ, McKenzie JE, Bossuyt PM, Boutron I, Hoffmann TC, Mulrow CD, et al. The PRISMA 2020 statement: an updated guideline for reporting systematic reviews. BMJ 2021;372:n71. doi: 10.1136/bmj.n71

For more information, visit: <http://www.prisma-statement.org/>

**Supplementary Table 6.** GRADE analysis of the primary and secondary outcomes

| **Quality assessment** | | | | | | | | | | | | **No of patients** | | **Effect** | | **Quality** | **Importance** |
| --- | --- | --- | --- | --- | --- | --- | --- | --- | --- | --- | --- | --- | --- | --- | --- | --- | --- |
|  |  |  |  |  |  |  |  |  |  |  |  |  |  |  |  |  |  |
| **No of studies** | | **Design** | | **Risk of bias** | **Inconsistency** | | **Indirectness** | | **Imprecision** | | **Other considerations** | **Intervention group** | **Control group** | **Relative (95% CI)** | **Absolute** |  |  |
| **Effectiveness of mindfulness yoga on depression (post-intervention) (Better indicated by lower values)** | | | | | | | | | | | | | | | | | |
| 9 | | randomized trials | | no serious risk of bias | serious^1^ | | no serious indirectness | | no serious imprecision | | reporting bias^2^ | 298 | 270 | - | SMD 0.53 lower (0.96 to 0.11 lower) | ⊕⊕OO LOW | CRITICAL |
| **Effectiveness of mindfulness yoga on anxiety (post-intervention) (Better indicated by lower values)** | | | | | | | | | | | | | | | | | |
| 2 | randomized trials | | | no serious risk of bias | no serious inconsistency | no serious indirectness | | serious^3^ | | reporting bias^2^ | | 30 | 27 | - | SMD 1.08 lower (1.64 to 0.52 lower) | ⊕⊕OO LOW | CRITICAL |
| **Effectiveness of mindfulness yoga on rumination (post-intervention) (Better indicated by lower values)** | | | | | | | | | | | | | | | | | |
| 3 | randomized trials | | | no serious risk of bias | no serious inconsistency | no serious indirectness | | serious^3^ | | reporting bias^2^ | | 119 | 104 | - | SMD 0.33 lower (0.89 lower to 0.23 higher) | ⊕⊕OO LOW | CRITICAL |
| **Effectiveness of mindfulness yoga on rumination (follow-up) (Better indicated by lower values)** | | | | | | | | | | | | | | | | | |
| 2 | randomized trials | | no serious risk of bias | | no serious inconsistency | no serious indirectness | | serious^4^ | | reporting bias^2^ | | 25 | 18 | - | MD 7.42 lower (5.16 to 3.56 lower) | ⊕⊕OO LOW | CRITICAL |

^1^ I^2^ is 82%

^2^ Due to the limited number of studies included under each outcome, there might be a publication bias.

^3^ The sample size is small (The total sample size < 400)

^4^ The sample size is too small (The sample of each group size ≤ 30)
